# Advance care planning knowledge, attitudes, and experiences among hospital healthcare professionals: A survey

**DOI:** 10.1017/S1478951526101874

**Published:** 2026-02-19

**Authors:** Anna Giulia Macchiarelli, Emma Capulli, Rabih Chattat, Marco Domenicali, Giusy Iorio, Barbara Lenzi, Marco Maltoni, Giacomo Neri, Chiara Peterle, Silvia Seclì, Valentina Sironi, Danila Valenti, Giovanni Ottoboni, Francesca Ingravallo

**Affiliations:** 1 Department of Medical and Surgical Sciences (DIMEC), Alma Mater Studiorum, University of Bologna, Bologna, Italy; 2 Department of Psychology “R. Canestrari,” Alma Mater Studiorum, University of Bologna, Bologna, Italy; 3 Department of Primary Health Care, Internal Medicine Unit addressed to Frailty and Aging, S. Maria delle Croci Hospital, AUSL Romagna, Ravenna, Italy; 4 Qualitative Research Unit, Azienda USL-IRCCS di Reggio Emilia, Reggio Emilia, Italy; 5 Medical Oncology, IRCCS Azienda Ospedaliero-Universitaria di Bologna, Bologna, Italy; 6 UOC Rete delle Cure Palliative, AUSL di Bologna, Bologna, Italy; 7 Haematology and Transplant Unit, Azienda Ospedaliera Policlinico Consorziale of Bari, Bari, Italy

**Keywords:** Shared care planning, healthcare providers, palliative care, Law 219/17, education

## Abstract

**Aim:**

To explore hospital healthcare professionals’ (HCPs) knowledge, attitudes, and experiences on advance care planning (ACP), comparing different professions and care specialties, in a country where ACP is formally regulated.

**Methods:**

An online survey involving HCPs from different care specialties involved in ACP working in Italian hospitals. Different tests were used for comparisons among HCPs.

**Results:**

We included responses from 724 HCPs (259 physicians, 86 residents, 339 nurses, 40 physiotherapists). Despite only 29.7% of participants having received education on ACP, the majority (75.5%) had heard of ACP and were aware of its key elements. The main misconceptions concerned legal aspects, while uncertainty regarding ACP practical implementation and correct timing were among the main reported barriers. Virtually all participants favored ACP, and 81.1% considered ACP part of their duty, but ACP is seldom offered to patients and is not always documented. Knowledge and attitudes toward ACP were similar across professional roles, while ACP education and discussion varied across specializations, with the highest levels reported by Palliative Care HCPs. In most specialties, a substantial overlap can be noted between levels of ACP education and ACP discussion among all HCPs, while higher levels of discussion were generally observed among physicians, though the magnitude of the gap between education and discussion levels differed across care specialties.

**Significance of results:**

Despite ad hoc regulation and HCPs’ favorable attitudes, the legal aspects of ACP remain poorly understood and ACP implementation in hospitals is still low. This study supports the need for clear procedures and for inclusion of ACP education and training in the core curricula of all HCPs, suggesting the need for studies integrating social sciences to explore specialty-specific barriers and facilitators to ACP. Due to their unique level of engagement in the process, palliative care HCPs may play a pivotal role in implementing hospital-based ACP.

## Introduction

Advance care planning (ACP) is defined as a process enabling people “to define goals and preferences for future medical treatment and care, to discuss these goals and preferences with family and healthcare providers, and to record and review these preferences if appropriate” (Rietjens et al. 2017). Hospital admission could provide both the motivation and a valuable opportunity for clinicians to introduce ACP (Mohan et al. 2021), but its implementation in hospitals seems to be generally low, though data on the actual frequency with which hospitalized patients are offered ACP discussions in different care specialties remain limited (Schichtel et al. 2019; Ashana et al. 2021; Martina et al. 2021; Westbye et al. 2023).

The formal regulation of ACP by Italian Law 219/17, which is in line with the above-mentioned ACP definition and international recommendations supported by the European Association for Palliative Care (Rietjens et al. 2017), provides a precious opportunity to investigate the topic consistently.

Law 219 of 2017, which entered into force on January 31st 2018, in Italy, regulated both advance directives (ADs) (in the law called “advance treatment dispositions”) and ACP (in the law called “shared care planning”). According to Law 219/17, ADs may be signed by any competent adult, while ACP can be implemented within the patient-physician relationship in the case of chronic disabling diseases or progressive conditions with poor prognosis. ACP must be documented in the patient’s medical record and can be updated at any time upon their request or the physician’s recommendation; the healthcare team is obligated to follow the plan in the case of the patient losing their decision-making capacity. While Law 219/17 attributes the main responsibility in ACP to physicians, non-medical healthcare professionals (HCPs) who are part of the healthcare team are involved in ACP according to their expertise.

To date, a few studies have investigated the perspective and experience of small groups of Italian physicians and nurses toward ACP, mainly with a qualitative approach, suggesting little ACP training, and a lack of standardized procedures and collaboration, with inconsistent implementation of ACP across care settings (Cipolletta and Reggiani 2021; Bombaci et al. 2024; Porteri et al. 2024).

The C.OP.E.R.NI.CO. (Knowledge, Opinions, and Experiences of Healthcare Professionals Regarding Shared care planning) survey aimed to investigate the knowledge, attitudes, and experiences regarding ACP on the part of HCPs from hospital units in which patients are most likely to need ACP discussions, including palliative care, comparing different professions. To fully implement ACP during hospital admission, indeed, all HCPs, including residents, should have adequate knowledge and attitudes toward ACP, and accurate knowledge of the provisions made by the law (Bryant et al. 2022). Moreover, the study aimed to compare levels of ACP education and discussion across different care specialties.

The results of this study may be useful to inform best practices and policy strategies to better integrate ACP in hospital clinical practice and for international comparisons.

## Methods

### Study design

This was a cross-sectional, self-administered, online survey. The protocol and manuscript followed the Strengthening the Reporting of Observational Studies in Epidemiology statement (Von Elm et al. 2007).

### Setting and participants

The survey was conducted in the Emilia Romagna region, located in northern Italy, in line with a previous study involving 12 Italian nursing homes in 2013–2015 (Ottoboni et al. 2019). Aiming at including medical areas that are presumably more involved in ACP, the study target population consisted of hospital HCPs working in the specialties of Anesthesia-Intensive Care, Cardiology, Gastroenterology, Geriatrics, Internal Medicine, Nephrology-Dialysis, Neurology, Oncology and Hematology, Pneumology, Rehabilitation, Surgery, and Palliative Care networks from different local health trusts.

### Data collection and recruitment strategy

Data was collected between October 2022 and February 2023 through the Qualtrics platform.

The first step of recruitment was to contact coordinators/directors of medical, non-medical, and psychological professions of the selected specialties and directors of the related Specialization Schools, in order to involve the medical residents. Coordinators/directors who accepted the invitation to collaborate in the study were asked to send information regarding the purposes and methods of the survey to all their HCPs via e-mail, along with a link to the website hosting the survey, with at least 1 reminder after 2 weeks. After reading the study information sheet, HCPs who agreed to participate could access the questionnaire.

### Questionnaire

A multidisciplinary team (comprising 3 experts in palliative care, 2 residents in oncology, 1 expert in legal medicine, 1 internist, 1 expert in psychogeriatrics, 1 psychologist, 1 cultural anthropologist, and 1 nurse) adapted the questionnaire used in a previous study (Ottoboni et al. 2019) in light of Law 219/17. The questionnaire was, in turn, adapted and translated (with permission) from the questionnaire developed by Yee and colleagues to survey renal HCPs in Singapore (Yee et al. 2011). The questionnaire was evaluated for face validity by a random sample of 16 HCPs who did not participate in the study, and small changes were made based on their feedback.

The final version of the questionnaire (available in Appendix), included five sections:

1. The first section collected information on age, gender, professional role, and length of professional experience. Participants were then asked whether they had ever heard of ACP: participants responding positively were led to section 2, while the others skipped this section and were led to section 3.
2. The second section included 9 questions assessing ACP knowledge through a true/false format.
3. The beginning of the third section provided a definition of ACP and ADs according to Law 219/17 (Box 1), and participants were then required to express their level of agreement on a 5-point Likert scale (from strongly disagree to strongly agree) with 19 statements concerning the perceived negative impact of ACP discussions on patients and families, perceived barriers to ACP discussions, perceived benefits of ACP discussions, and views on patient autonomy (1 question was not included in the analysis).
4. The fourth section investigated participants’ personal experience with ADs, and their working experiences with ADs and ACP during the past working year and whether they considered ACP part of their duties.
5. The fifth section investigated characteristics of the participants’ main specialty, their experience and preferences concerning ACP education and education on palliative care, their opinion as to how useful they considered ACP for the patients in their care, and how important their personal beliefs were in their professional practice with regard to ACP. Finally, 4 questions focused on the healthcare organization that the participants worked for: whether Law 219/17 was implemented by specific procedures, whether, in their opinion, there were barriers to ACP, and their insight on barriers and possible facilitators (open-ended questions).

Box 1. **Definitions of ACP and advance directives provided to the survey participants**

ACP refers to the process based on dialogue that involves discussing with the patient and those close to them the possible progression of the illness, realistic expectations regarding quality of life, available treatments, and palliative care options. The aim is to allow the patient to express their preferences regarding the proposed care plan and their future care, including the possible appointment of a fiduciary. Once documented in the medical records, ACP is binding for the entire healthcare team in the event that the patient is no longer able to express their wishes.

Advance directives (ADs) refer to the document through which any individual – regardless of their current health status – can express their wishes regarding medical assessments and treatments, and designate a fiduciary to represent them in the event of future incapacity. ADs must be filed with a notary or with designated offices that submit them to the national ADs registry.

### Ethics

The study was approved by the Committee of Bioethics of the University of Bologna (Ref. Num. 0240037 achieved by 10/10/2022). Anonymity and confidentiality for participants were guaranteed: the questionnaire did not include questions that could allow direct or indirect identification of participants, and did not include information concerning their healthcare facility or affiliation. The system did not record any compiler data (e.g., IP addresses). The University’s personal data processing notice for non-nominative surveys according to GDPR was provided with the survey’s informed consent.

### Statistical analysis

Descriptive statistics are reported in percentages (%) and number of participants, and mean and standard deviation are reported in the case of continuous variables.

Chi-squared test and Kruskal–Wallis test were used for comparisons among HCPs for categorical and continuous variables, respectively. A *p*-value of <0.05 was considered significant.

Statistical analyses were performed using Stata SE 17.0.

## Results

In total, 1230 people accessed the survey, and 886 answered all the questions; 162 answers were excluded as the participant reported not being directly involved in patient care (e.g., working as a manager, teacher, technician, or working from home) or working in areas of specialization that were not addressed by the survey (e.g., home care, ambulance service). Answers were also excluded when the number of HCPs from one profession was too small for analysis (e.g., 8 psychologists, 2 dieticians).

The final population comprised 724 HCPs: 259 physicians (35.8%), 86 residents (11.9%), 339 nurses (46.8%), and 40 physiotherapists (5.5%).

### Participants’ characteristics

As shown in Table 1, physicians, residents, nurses, and physiotherapists significantly differed in terms of personal and professional characteristics.

**Table 1.** Participants’ personal and professional characteristics (*N* = 724)

		All (724)	Physicians (259)	Residents (86)	Nurses (339)	Physioth. (40)	*p*
Gender % (*n*)	Male	26.2 (190)	36.7 (95)	32.6 (28)	18.0 (61)	15.0 (6)	<0.001
	Female	73.6 (533)	63.3 (164)	67.4 (58)	81.7 (277)	85.0 (34)	
	Other	0.1 (1)	0.0 (0)	0.0 (0)	0.3 (1)	0.0 (0)	
Age range (years) % (*n*)	<30	20.9 (151)	2.7 (7)	79.1 (68)	21.2 (72)	10.0 (4)	<0.001
	31–40	28.7 (208)	33.2 (86)	19.8 (17)	28.3 (96)	22.5 (9)	
	41–50	24.3 (176)	28.6 (74)	1.2 (1)	26.8 (91)	25.0 (10)	
	51–60	20.3 (147)	22.4 (58)	0.0 (0)	22.4 (76)	32.5 (13)	
	>60	5.8 (42)	13.1 (34)	0.0 (0)	1.2 (4)	10.0 (4)	
Main specialty % (*n*)	Anesthesia-Intensive Care	25.7 (186)	23.9 (62)	24.4 (21)	30.4 (103)	0.0 (0)	<0.001
	Cardiology	3.0 (22)	3.1 (8)	0.0 (0)	3.5 (12)	5.0 (2)	
	Gastroenterology	4.0 (29)	3.9 (10)	13.9 (12)	2.1 (7)	0.0 (0)	
	Geriatrics	7.0 (51)	9.6 (25)	7.0 (6)	5.6 (19)	2.5 (1)	
	Internal Medicine	16.3 (118)	19.3 (50)	27.9 (24)	12.7 (43)	2.5 (1)	
	Nephrology-Dialysis	3.7 (27)	3.9 (10)	0.0 (0)	5.0 (17)	0.0 (0)	
	Neurology	2.9 (21)	6.2 (16)	3.5 (3)	0.3 (1)	2.5 (1)	
	Oncology and Hematology	5.9 (43)	5.4 (14)	13.9 (12)	5.0 (17)	0.0 (0)	
	Palliative Care	8.6 (62)	7.3 (19)	0.0 (0)	12.7 (43)	0.0 (0)	
	Pneumology	4.3 (31)	4.2 (11)	4.6 (4)	4.4 (15)	2.5 (1)	
	Rehabilitation	8.6 (62)	5.4 (14)	3.5 (3)	3.8 (13)	80 (32)	
	Surgery	9.9 (72)	7.7 (20)	1.2 (1)	14.4 (49)	5.0 (2)	
Completed their studies before 2018 % (*n*)	Yes	75.3 (545)	83.0 (215)	4.6 (4)	84.7 (287)	97.4 (39)	<0.001
Had some education on ACP % (*n*)	During degree						<0.001
	*Theoretical*	9.5 (69)	1.9 (5)	20.9 (18)	12.9 (44)	5.0 (2)	
	*Theoretical + practical*	1.2 (9)	1.5 (4)	0.0 (0)	1.5 (5)	0.0 (0)	
	Post-degree						
	*Theoretical*	11.7 (85)	12.7 (33)	5.8 (5)	12.7 (43)	10.0 (4)	
	*Theoretical + practical*	7.2 (52)	9.3 (24)	0.0 (0)	7.7 (26)	5.0 (2)	
	No	70.3 (509)	74.5 (193)	73.3 (63)	65.2 (221)	80.0 (32)	
Had some education on palliative care % (*n*)	Yes, structured program	6.3 (46)	6.2 (16)	3.5 (3)	3.4 (25)	5.0 (2)	<0.001
	Yes, hours/days	42.5 (308)	35.1 (91)	31.4 (27)	52.8 (179)	27.5 (11)	
	No	51.1 (370)	58.7 (152)	65.1 (56)	39.8 (135)	67.5 (27)	
Interested in exploring ACP further % (*n*)	Yes, training	5.3 (38)	4.6 (12)	3.5 (3)	6.2 (21)	5.0 (2)	0.215
	Yes, theory	14.2 (103)	16.6 (43)	10.5 (9)	13.3 (45)	15.0 (6)	
	Yes, theory + training	75.7 (548)	75.3 (195)	84.9 (73)	73.5 (249)	77.5 (31)	
	No	4.8 (35)	3.5 (9)	1.2 (1)	7.1 (24)	2.5 (1)	

ACP = advance care planning.

Most participants were female (73.6%), were between 30 and 60 years old, and had completed their studies before 2018, except for residents, who were, for the most part, younger than 30 years old (79.1%) and had completed their studies after 2018. Participants were mostly physicians, residents, and nurses in Anesthesia-Intensive Care and Internal Medicine, while Rehabilitation was the main specialty for the physiotherapists. Palliative Care was the main specialty for 9% of all HCPs and for 7% of physicians.

Regarding ACP education and training, 9.5% of participants (ranging from 1.9% of physicians to 20.9% of residents) received ACP theoretical education during the university degree, while 1.2% (ranging from 0.0% of residents to 1.5% of both physicians and nurses) received both theoretical and practical education during their university degree, 11.7% (ranging from 5.8% of residents to 12.7% of physicians) had some post-degree theoretical education, while 7.2% (ranging from 0.0% of residents to 9.3% of physicians) reported post-degree theoretical and practical education; 70.3% of HCPs (ranging from 65.2% of nurses to 80.0% of physiotherapists) did not receive any ACP education/training.

A total of 48.8% of participants (ranging from 32.5% of physiotherapists to 56.2% of nurses) reported receiving some education on palliative care, mainly consisting of courses lasting for a few hours or days.

Almost all participants were interested in exploring the topic of ACP further, with the vast majority (from 73.5% of nurses to 84.9% of residents) preferring both theoretical education and practical training, without significant differences across professions.

Other results are provided in Supplementary Table 1.

### Advance care planning knowledge

Overall, 75.5% of participants reported having heard of ACP and completed the knowledge section: 77.6% of physicians, 66.3% of residents, 77.0% of nurses, and 70.0% of physiotherapists (*p* = 0.130).

The questions with the higher frequencies of correct responses (93.0–98.9%) were those concerning ACP goals, conditions for which ACP is appropriate, the need for good communication skills, and the possibility of reviewing ACP if necessary. The questions with the lower rate of correct responses were those concerning legal aspects of ACP, including means of documentation for ACP (for which correct answers ranged from 26.3% of residents to 52.7% of physicians, *p* < 0.001), the need to comply with ACP in case of patient incapacity (77.7% of correct answers overall, without significant differences).

Answers to the knowledge section are reported in Supplementary Table 2.

### Attitudes toward advance care planning

Table 2 shows the percentage of participants who strongly agreed/agreed with each item in the attitude section.

**Table 2.** Percentage of participants who strongly agreed/agreed with each statement of the attitudes section (*N* = 724)

Statement	All (724)	Physicians (259)	Residents (86)	Nurses (339)	Physioth. (40)	*p*
I favor the use of ACP	97.2 (704)	96.5 (250)	96.5 (83)	98.2 (333)	95.0 (38)	0.449
*Perceived negative impact of ACP on patient and family*						
I worry that discussing ACP could upset the patient	21.1 (153)	23.5 (61)	20.9 (18)	19.2 (65)	17.5 (7)	0.573
Discussing ACP will make patients lose hope	5.0 (36)	4.2 (11)	5.8 (5)	5.3 (18)	5.0 (2)	0.919
If death is discussed, the patient may wish to die	8.7 (63)	5.0 (13)	7.0 (6)	12.4 (42)	5.0 (2)	0.011
If HCPs discuss ACP with the patient, the family may blame them for the patient’s choices	18.5 (134)	16.2 (42)	19.8 (17)	20.6 (70)	12.5 (5)	0.395
Discussing ACP is advocating euthanasia	2.6 (19)	0.8 (2)	2.3 (2)	4.4 (15)	0.0 (0)	0.031
*Perceived barriers to ACP discussion*						
I don’t feel I have the emotional strength to support the patient through ACP discussion	10.6 (77)	8.9 (23)	10.5 (9)	12.1 (41)	10.0 (4)	0.655
I don’t have the skills to discuss ACP with the patient	19.2 (139)	11.2 (29)	32.6 (28)	20.1 (68)	35.0 (14)	<0.001
I don’t have the time for ACP discussions	17.4 (126)	12.7 (33)	18.6 (16)	20.9 (71)	15.0 (6)	0.068
The operational implementation of ACP is not clear	37.8 (274)	33.6 (87)	59.3 (51)	36.3 (123)	32.5 (13)	<0.001
Most of our local patients are not ready for ACP discussion	31.2 (226)	28.2 (73)	33.7 (29)	33.9 (115)	22.5 (9)	0.269
Most HCPs in my local health organization are not ready for ACP discussion	37.6 (272)	35.1 (91)	39.5 (34)	39.5 (134)	32.5 (13)	0.617
It is difficult to understand the right time to implement ACP	36.0 (261)	37.1 (96)	52.3 (45)	39.7 (104)	40.0 (16)	<0.001
*Perceived benefits of ACP discussion*						
ACP helps in the care of patients when they are seriously ill	88.5 (641)	95.7 (248)	93.0 (80)	87.3 (296)	85.0 (34)	0.002
For family members, ACP can decrease the burden of decisions	90.1 (652)	91.9 (238)	93.0 (80)	92.3 (313)	90.0 (36)	0.944
ACP allows patients to have a sense of control over their life	92.1 (667)	91.9 (238)	91.9 (79)	85.2 (289)	87.5 (35)	0.059
*Views on patient’s autonomy*						
Prolonging life is more important than honoring a patient’s request to refuse life-sustaining treatment	4.6 (33)	3.5 (9)	1.2 (1)	5.9 (20)	7.5 (3)	0.159
It is important for patients to be able to influence their future treatment should they lose competence in decision making	94.9 (687)	96.1 (249)	93.0 (80)	94.7 (321)	92.5 (37)	0.583

ACP = advance care planning; HCP = healthcare professional.

Almost all HCPs (97.2%) agreed with the statement “I favor the use of ACP.”

Concerning the perceived negative impact of ACP discussion, the 2 statements with the highest rates of agreement were the concern that discussing ACP might upset the patient, and that the family may blame HCPs for the patient’s choices (21.1% and 18.5%, without significant differences among professions).

Regarding perceived barriers to ACP, statements with highest rate of agreement were those concerning the lack of clarity regarding operational implementation of ACP (37.8%) and the difficulty in understanding the right time for ACP (36.0%), with a significantly higher rate of residents agreeing with these statements (59.3% and 52.3%, respectively); another perceived barrier was the unreadiness of healthcare providers and patients for ACP (overall rate of agreement of 37.6% and 31.2%, respectively, without significant differences across professions).

Finally, almost all participants (88.5–94.9%) agreed with the benefits of ACP and the importance of patient autonomy.

### Experience with advance directives and advance care planning

As described in Table 3, 7.6% of participants had completed ADs for themselves, and 6.3% reported having a family member with ADs (*p* = 0.413).

**Table 3.** Experiences with advance directives and ACP (*N* = 724)

		All (724)	Physicians (259)	Residents (86)	Nurses (339)	Physioth. (40)	*p*
Have you cared for a patient who has had advance directives? % (*n*)	Yes	33.1 (240)	44.0 (114)	32.6 (28)	25.7 (87)	27.5 (11)	<0.001
Have you cared for a patient who has had an advance care plan? % (*n*)	Yes	30.8 (223)	44.0 (114)	18.6 (16)	22.7 (77)	40.0 (16)	<0.001
Do you consider ACP as part of your duty? % (*n*)	Yes	81.1 (587)	88.0 (228)	68.6 (59)	81.4 (276)	60.0 (24)	<0.001
	Don’t know	14.8 (107)	8.1 (21)	23.3 (29)	15.6 (53)	32.5 (13)	
	No	4.1 (30)	3.9 (10)	8.1 (7)	2.9 (20)	7.5 (3)	
Have you personally discussed/participated in ACP discussion? % (*n*)	Yes	34.5 (250)	57.1 (148)	24.4 (21)	21.2 (72)	22.5 (9)	<0.001
Do you consider ACP useful/very useful for patients cared for? % (*n*)	Yes	92.4 (669)	94.6 (245)	94.2 (81)	89.9 (305)	95.0 (38)	0.145
Has Law 219/17 been implemented in your healthcare organization with specific procedures? % (*n*)	Yes	22.8 (165)	29.3 (76)	10.5 (9)	20.3 (69)	27.5 (11)	<0.001
	Don’t know	51.7 (374)	45.6 (118)	75.6 (65)	49.8 (169)	55.0 (22)	
	No	25.5 (185)	25.1 (65)	13.9 (12)	29.8 (101)	17.5 (7)	
Do you believe there are obstacles to ACP in your healthcare organization? % (*n*)	Yes	53.6 (388)	46.2 (118)	65.1 (56)	58.4 (198)	40.0 (16)	<0.001

Ads = advance directives; ACP = advance care planning.

Regarding professional experiences during the past year, 33.1% and 30.8% of HCPs reported having cared for a patient with ADs or ACP, respectively, with physicians reporting these experiences more often than other HCPs (44.0%, *p* < 0.001).

Physicians were those who more often recognized ACP as part of their duties (88.0%), followed by nurses (81.4%), while the highest level of uncertainty on the issue was reported by physiotherapists (32.5%) and residents (23.3%) (*p* < 0.001).

**Figure 1.**
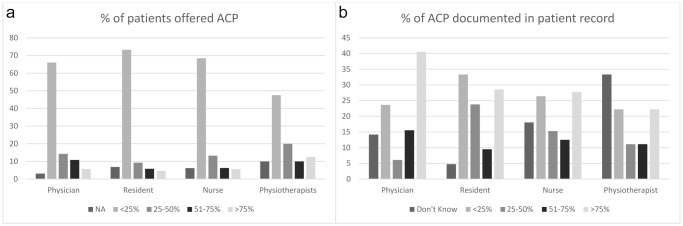
(a) Estimation of the percentage of patients to whom ACP discussion was offered (considering only patients for whom ACP was appropriate) (<25% include the answer “None”) and (b) percentage of documented ACP in patient records (<25% includes the answer “Never”).

Figure 1a shows an estimation of the percentage of patients to whom an ACP discussion was offered (considering only patients for whom ACP was appropriate). In total, 49.4% of participants indicated that ACP was offered to less than 25% of patients, while 5.9% indicated that it was offered to more than 75% of patients.

Overall, 34.5% of participants (57.1% of physicians vs. 21.2–24.4% of other HCPs, *p* < 0.001) reported having personally discussed/participated in ACP discussion with the patient during the past year, and Figure 1b shows their responses in terms of how often ACP was documented in patient records: 35.6% indicated that ACP was recorded in fewer than 50% of cases, while 35.2% that it was recorded in over 75% of cases.

Based on the characteristics of their patients, 92.4% of participants (ranging from 90% of nurses to 95% of physiotherapists) considered ACP a useful/very useful tool (*p* = 0.145).

With reference to their healthcare organization, 51.7% of participants (ranging from 45.6% of physicians to 75.6% of residents) did not know whether ACP was implemented with specific procedures, while 22.8% (ranging from 10.5% of residents to 29.3% of physicians) answered that ACP had been implemented (*p* < 0.001). Finally, 53.6% of HCPs (ranging from 40.0% of physiotherapists to 65.1% of residents) believed that there were obstacles to ACP within the context of their healthcare organization (*p* < 0.001).

Other results concerning the ACP discussion are reported in Supplementary Table 3.

### Advance care planning education and discussion across different care specialties

**Figure 2.**
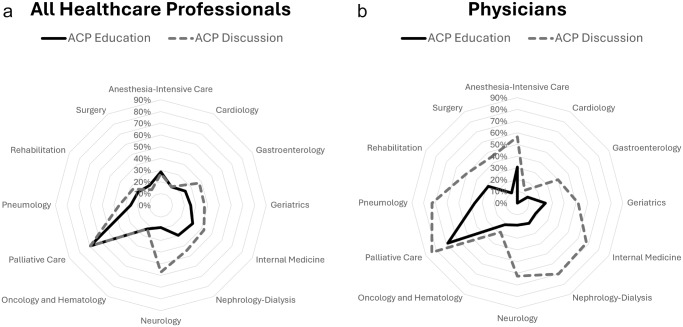
Levels of ACP education and of ACP discussion across care specialties: (a) all HCPs and (b) physicians only.

Figure 2a shows levels of ACP education (either theoretical only or both theoretical and practical) and of ACP discussion during the past year reported by HCPs across the different care specialties. Figure 2b concerns physicians only.

ACP levels of education ranged from 18% reported in Cardiology to 69% reported in Palliative Care among HCPs overall, and from 0% in Cardiology to 68% in Palliative Care considering physicians only.

ACP discussion levels ranged from 15% in Surgery to 69% in Palliative Care among HCPs overall and from 13% in Cardiology to 84% in Palliative Care among physicians.

Among all the HCPs, a substantial overlap between education and discussion levels can be noted in most care specialties (gap ranging from −4% in Surgery to +14% in Gastroenterology and Nephrology-Dialysis), but in Neurology (+38% in favor of discussion). Considering physicians only, across all care specialties, discussion levels exceeded education levels, with the smallest gaps in Oncology (+8%) and Cardiology (+13%), and the largest gaps in Internal Medicine (+50%), Nephrology-Dialysis (+50%), and Neurology (+44%).

## Discussion

Investigating the perspective of hospital HCPs is essential to understand the state of the art of ACP in hospital clinical practice and which factors should be considered to increase its implementation.

The main results of this study are that, with the exception of those working in Palliative Care, only a minority of hospital HCPs had some education or personal experience with ACP discussion. Nevertheless, most HCPs were aware of ACP and had a sound knowledge of its key elements, with the exception of ACP legal requirements. ACP is viewed with favor and considered by the majority of HCPs as part of their duty. However, ACP is seldom offered to patients, and is not always documented, the main barriers being a lack of clarity regarding its practical implementation and correct timing. Overall, physicians, residents, nurses and physiotherapists showed similar knowledge and attitudes toward ACP, but we found inter-specialty differences. Comparing education and discussion levels across care specialties, we observed an overlap among all HCPs, while higher levels of discussion were generally observed among physicians, though the magnitude of the gap differed across care specialties.

Our results show a considerably greater awareness of ACP compared with a previous study carried out in Italian nursing homes (in which 29.7% of participants reported having heard of ACP) (Ottoboni et al. 2019). However, still only a minority of hospital HCPs, including residents, had some education, mainly theoretical, and experience with ACP. This finding is in line with studies carried out in other countries (Chandar et al. 2017; Schichtel et al. 2019; Ashana et al. 2021; Martina et al. 2021) and indicates that, despite the legal regulation of ACP, both university and healthcare systems underwent delays in integrating legislative changes into education and hospital practice. ACP seems to be more integrated into the curriculum and clinical practice of only Palliative Care HCPs, in line with their vocation in managing sensitive conversations and exploring patients’ end-of-life preferences (Ferrell et al. 2018).

Despite their familiarity with the key elements of ACP, in line with studies carried out in other countries and care settings (Yee et al. 2011; Kermel-Schiffman and Werner 2017; Shepherd et al. 2018; Hooper et al. 2020; Martina et al. 2021), HCPs were less knowledgeable regarding legal aspects, suggesting an ongoing challenge in bridging the gap between legal and medical perspectives in ACP (Hooper et al. 2020). The main misconceptions concerned ACP documentation and its binding value and may represent a concrete barrier to ACP implementation in clinical practice, since confusion concerning how to implement the law in practice creates uncertainty (Ke et al. 2019; Maffoni et al. 2020; Mohan et al. 2021).

We found that only around one third of HCPs (44% of physicians) had cared for a patient with ACP during the past year, and that, in line with studies carried out in other countries, most hospitalized patients were not offered ACP discussion (Glaudemans et al. 2015; Knight et al. 2020; Ashana et al. 2021). A gap between favorable attitudes of HCPs toward ACP and limited ACP implementation was found by previous studies carried out since Law 219/17 entered into force. HCPs viewed ACP as a useful tool for aligning the care provided with patients’ preferences and improving communication between healthcare providers, patients, and families; however, these favorable attitudes did not result in consistent implementation of ACP in clinical practice, mainly due to a lack of knowledge and training among HCPs, a lack of standardized procedures, little awareness on the part of the general population, and cultural or religious influences (Maffoni et al. 2020; Testoni et al. 2020; Cipolletta and Reggiani 2021; Porteri et al. 2024). At an international level, several barriers to ACP implementation in hospitals have been reported, including cultural factors, role ambiguity, lack of time, discomfort with end-of-life themes, and concerns over patients’ reactions (Hsieh et al. 2019; Ashana et al. 2021; Martina et al. 2021; Westbye et al. 2023). In our study, half of the participants indicated that barriers to ACP existed in their healthcare organization, and among the main barriers were difficulty understanding the right time for ACP discussion and a lack of clear procedures for its operational implementation, which may be worsened by the lack of knowledge of existing procedures at the hospital level reported by half of the HCPs.

Despite the clear provisions of Law 219/17, there are no national guidelines for ACP in Italy, and it was argued that much is left to the “good will” of the single HCP (Cipolletta and Reggiani 2021). On the contrary, the ACP application requires institutional commitment and well-defined procedures to embed ACP into daily workflows (Gilissen et al. 2018, 2019). Professional guidelines and hospital procedures should also address ACP timing. With this regard, even if it remains challenging to determine a clear threshold for discussions to be initiated (Knight et al. 2020; Westbye et al. 2023), recent studies suggest that patients would prefer ACP conversations to be initiated earlier than in current practice (Zhu et al. 2025).

One of the main aims of C.OP.E.R.NI.CO. was to compare HCPs with different professional roles. We found that, despite significant differences in almost all personal and professional characteristics, including ACP education and experiences, there were few differences concerning ACP knowledge and attitudes across professions. Moreover, even with more uncertainties among medical residents and physiotherapists, very few participants did not recognize ACP as part of their professional duty. This may suggest a widespread acceptance of the ACP construct among hospital HCPs who are in direct contact with patients, while differences concerning experiences with ACP may reflect different role-based responsibilities, differences in clinical tasks, and unequal exposure to ACP education and discussions. Physicians reported the highest level of ACP discussion (57.1%), while only 21.2% of nurses and 22.5% of physiotherapists reported having participated in ACP discussion during the past year, respectively. These findings may reflect the prominent role that Law 219/17 attributes to the physician as the main ACP provider, resulting in a low level of involvement of non-medical HCPs in ACP. Moreover, with regard to nurses, it is possible that our findings were influenced by the large proportion of nurses working in Anesthesia-Intensive Care and Surgery, including the operating room, who may have had little opportunity to discuss ACP despite their greater exposure to ACP and palliative care education. This hypothesis could also explain why, in our survey, nurses were those who were less likely to consider ACP useful for the patients in their care. On the contrary, the results concerning physiotherapists were in line with their lower levels of ACP and palliative care education and the highest proportion of uncertainty regarding whether ACP is part of their professional duty. Future studies are needed to better address this topic.

Findings regarding residents warrant a separate discussion. To our knowledge, this is the first study comparing residents, physicians, and other HCPs. We found that residents reported less ACP training, had more legal misconceptions, were more likely to report inadequate skills, perceived obstacles to ACP in their health organizations, as well as uncertainty regarding the existence of specific procedures in their healthcare organization. These findings support the need for both specific training, including ethical reasoning skill development and supervisor feedback, and structural reforms to better integrate ACP into residents’ clinical practice (Fassier et al. 2021; Dias et al. 2023; Bombaci et al. 2024).

Regarding comparison among care specialties, the substantial inter-specialty differences in ACP could be attributed to several reasons, including variations in patient characteristics, in the participants’ clinical roles, or in the communication training backgrounds of the respondents. Considering that we surveyed care specialties in which patients are presumably most likely to need ACP, the variability we found is striking.

Considering hospital HCPs overall, we observed an overlap between education and discussion levels that supports the link between ACP education and ability to engage with ACP in different care specialties (Chandar et al. 2017; Chan et al. 2019; Yamamoto et al. 2022; Sakamoto et al. 2023; Zhang et al. 2025).

Among physicians, we observed a consistent gap in favor of ACP discussion over education, which was especially evident in some care specialties, such as Internal Medicine, in which frail older adults are the main population of patients, Nephrology-Dialysis and Neurology. This suggests that when ACP is required, many physicians adopt informal or experiential approaches. Quality assessment of both the process and the outcomes is necessary to understand the implications of these approaches.

Conversely, in specialties such as Cardiology, Oncology and Hematology, physicians reported both absent or low levels of ACP education and low levels of ACP discussion. We may speculate that additional challenges to ACP associated with specific conditions, including assessing decisional capacity and managing unpredictable disease trajectories or limited continuity of care (Jabbarian et al. 2018; Schichtel et al. 2019; Ladin et al. 2021; Guccione et al. 2023; Naik et al. 2025), may differently influence the implementation process across care specialties. In order to gain better insight into ACP specialty-specific barriers and facilitators, future studies comparing specialties should incorporate narrative, phenomenological, and ethnographic approaches (De Vleminck and Van den Block 2023).

Highlighting as it does the unique level of engagement in ACP of HCPs workin in Palliative Care, this study suggests that they may act as catalysts for the development and implementation of hospital-based ACP, helping to translate broadly favorable attitudes toward ACP into more consistent clinical practice, through interventions at different levels. Since palliative care skills are among the main enablers in aiding discussions on ACP (Guccione et al. 2023), palliative care HCPs may have a primary role in ACP training (Chandar et al. 2017), contributing to interprofessional education from undergraduate curricula onwards to strengthen communication and collaboration (Ke et al. 2019). Moreover, they may have a pivotal role in facilitating multidisciplinary meetings of healthcare teams, patients, and families, particularly in complex cases. Finally, based on their greater experience with ACP in the hospital context, palliative care HCPs may have a leading role at the institutional level in the development of local ACP procedures, documentation pathways, and organizational cultures to integrate ACP into routine hospital practice.

The strengths of this study include the high number of participants involved, the inclusion of both physicians and other HCPs, and the variety of care specialties surveyed. However, several limitations of the study should be acknowledged. As this study was conducted in Northern Italy, it does not account for the potential regional differences regarding ACP or palliative care education suggested in previous studies (Bombaci et al. 2024). The large number of participants working in Anesthesia-Intensive Care could have negatively affected the proportion of HCPs who had the opportunity to discuss ACP in clinical practice, since these HCPs often come into contact with patients during a single preoperative consultation or work with patients who are sedated or unable to discuss ACP. Moreover, the use of an anonymous, self-administered online survey, while effective in reaching a large number of participants, may have introduced a self-selection bias, potentially attracting HCPs with a pre-existing interest in or awareness of ACP. A self-selection bias is inherent to any survey of this nature (Hsieh et al. 2019; Dias et al. 2023; Sakamoto et al. 2023) and may have influenced the high proportion of HCPs with positive attitudes toward ACP, increasing the gap between attitudes and practice. Future research could specifically focus on the perspective of HCPs who are less favorable to ACP. Moreover, while the anonymity and confidentiality assured by the survey design aim to reduce any social desirability bias, they do not eliminate this risk (Chandar et al. 2017; Hsieh et al. 2019; Ke et al. 2019; Dias et al. 2023). Including 4 distinct, well-defined professional groups in the analysis was useful in order to provide a wider picture of different HCPs’ perspectives and experiences with ACP. The inclusion of physiotherapists, in particular, offered original insight. However, due to an insufficient number of respondents from certain professions, which may be greatly involved in ACP (e.g., psychologists), it was necessary to exclude them from the analysis, preventing us from obtaining a more complete picture. Finally, the cross-sectional design does not allow for causal inferences.

Taking these limitations into account, the C.OP.E.R.NI.CO. study provided a picture of ACP in hospital practice in a country where it is formally regulated by law. A large multi-professional population, including little-studied groups such as medical residents and physiotherapists, was surveyed across multiple care specialties, integrating knowledge, attitudes, and experiences that may help to address the issue of why ACP implementation in the hospital setting remains limited.

In summary, this study shows that formal regulation of ACP by law is not *per se* sufficient to reach desirable levels of ACP education and practice among hospital HCPs, highlighting the importance of investigating HCPs’ attitudes and practices in parallel, as favorable views toward ACP do not always translate into its implementation. Specific ACP theoretical education and practical training are needed and would be welcomed by most hospital HCPs. Special attention should be given to conveying legal concepts and to training residents. Moreover, our results indicate the need not only to develop local procedures (including clear documentation procedures) but also to ensure that they are disseminated adequately. Finally, showing the existence of substantial differences across hospital care specialties, this study suggests the existence of specialty-specific barriers and facilitators that may differentially operate in addition to individual, professional, organizational, and cultural factors in affecting ACP implementation in hospital clinical practice. In this composite scenario, palliative care HCPs may play a pivotal role in helping to translate broadly favorable attitudes toward ACP into more consistent clinical practice through education, clinical leadership, and institutional support and guidance in implementing hospital-based ACP.

## Supporting information

10.1017/S1478951526101874.sm001 Macchiarelli et al. supplementary material 1 Macchiarelli et al. supplementary material

10.1017/S1478951526101874.sm002 Macchiarelli et al. supplementary material 2 Macchiarelli et al. supplementary material

## Supplementary material

The supplementary material for this article can be found at https://doi.org/10.1017/S1478951526101874.
