# Supplementary material for: Advance care planning knowledge, attitudes, and experiences among hospital healthcare professionals: A survey
Source: Palliat Support Care. 2026 Feb 19;24:e61. doi: 10.1017/S1478951526101874 (PMC13166266; doi:10.1017/S1478951526101874)
Supplement: Macchiarelli et al. supplementary material 1 — Macchiarelli et al. supplementary material [file S1478951526101874sup001.docx]

**C.OP.E.R.NI.CO. (Knowledge, Opinions, and Experiences of Healthcare Professionals Regarding Advance care planning) study**

**Questionnaire**

**SECTION 1**

How old are you?

o Under 30

o From 31 to 40

o From 41 to 50

o From 51 to 60

o Over 60

How do you identify?

o Man

o Woman

o Other __________________________________________________

What is your profession?

o Physiotherapist

o Nurse

o Physician

o Resident

o Psychologist

o Other / Specify __________________________________________________

How long have you been in your profession? ___ years

Have you ever heard of Advance Care Planning?

o Yes

o No

If Yes, please go to Section 2. If No, please skip Section 2 and go straight to Section 3.

**SECTION 2**

**Please indicate whether each of the following statements is true or false**

|  | True | False |
| --- | --- | --- |
| 2.1. Advance care planning may be carried out in relation to the progression of chronic or disabling diseases or diseases with poor prognosis | ① | ② |
| 2.2. Advance care planning can be done without patient signing up to an advance directive | ① | ② |
| 2.3. Within advance care planning the patient expresses their wills regarding the care plan proposed and may indicate the name of a fiduciary | ① | ② |
| 2.4. The healthcare team must always adhere to the advance care planning should the patient be in a condition of incapacity | ① | ② |
| 2.5. Advance care planning has no legal value | ① | ② |
| 2.6. Good communication skills are essential for advance care planning discussion | ① | ② |
| 2.7. Advance care planning must be documented as a public act or as a notarized private agreement | ① | ② |
| 2.8. Patient consent is necessary to involve family/caregivers in advance care planning | ① | ② |
| 2.9. Advance care planning can be updated as the disease progresses upon the patient’s request or the physician’s recommendation | ① | ② |

**SECTION 3**

In this questionnaire, and in accordance with Law No. 219/2017, which regulates both advance care planning and advance directives (AD) in Italy, the following definitions apply:

-Advance care planning refers to the process based on dialogue that involves discussing with the patient and those close to them the possible progression of the illness, realistic expectations regarding quality of life, available treatments, and palliative care options. The aim is to allow the patient to express their preferences regarding the proposed care plan and their future care, including the possible appointment of a fiduciary. Once documented in the medical records, advance care planning is binding for the entire healthcare team in the event that the patient is no longer able to express their wishes.

-Advance directives (ADs) refer to the document through which any individual—regardless of their current health status—can express their wishes regarding medical assessments and treatments, and designate a fiduciary to represent them in the event of future incapacity. ADs must be filed with a notary or with designated offices that submit them to the national ADs registry.

**Please indicate how much you agree, in general, with each of the following statements about advance care planning**

|  | **Strongly Disagree** | **Disagree** | **Undecided** | **Agree** | **Strongly Agree** |
| --- | --- | --- | --- | --- | --- |
| 3.1. I favor the use of advance care planning | ① | ② | ③ | ④ | ⑤ |
| 3.2. I worry that discussing advance care planning could upset the patient | ① | ② | ③ | ④ | ⑤ |
| 3.3. Discussing advance care planning will make the patients lose hope | ① | ② | ③ | ④ | ⑤ |
| 3.4. I don’t feel I have the emotional strength to support the patient through advance care planning discussion | ① | ② | ③ | ④ | ⑤ |
| 3.5 I don’t have the skills to discuss advance care planning with the patient | ① | ② | ③ | ④ | ⑤ |
| 3.6. Most of our local patients are not ready for advance care planning discussion | ① | ② | ③ | ④ | ⑤ |
| 3.7. Most healthcare professionals in my local health organization are not ready for advance care planning discussion | ① | ② | ③ | ④ | ⑤ |
| 3.8. If healthcare professionals discuss advanced care planning with the patient, the family may blame them for the patient’s choices | ① | ② | ③ | ④ | ⑤ |
| 3.9. I don’t have the time for advance care planning discussions | ① | ② | ③ | ④ | ⑤ |
| 3.10. The operational implementation of advance care planning is not clear | ① | ② | ③ | ④ | ⑤ |
| 3.11. If death is discussed, the patient may wish to die. | ① | ② | ③ | ④ | ⑤ |
| 3.12. Discussing advance care planning is advocating euthanasia | ① | ② | ③ | ④ | ⑤ |
| 3.13. Advance care planning helps in the care of patients when they are seriously ill | ① | ② | ③ | ④ | ⑤ |
| 3.14. For family members, advance care planning can decrease the burden of decisions | ① | ② | ③ | ④ | ⑤ |
| 3.15. 1. Advance care planning allows patients to have a sense of control over their life | ① | ② | ③ | ④ | ⑤ |
| 3.16. Prolonging life is more important than honoring a patient’s request to refuse life-sustaining treatment. | ① | ② | ③ | ④ | ⑤ |
| 3.17. It is important for patients to be able to influence their future treatment should they lose competence in decision making. | ① | ② | ③ | ④ | ⑤ |
| 3.18. It is not possible to discuss advance care planning if the patient is not fully aware of their clinical condition | ① | ② | ③ | ④ | ⑤ |
| 3.19. It is difficult to understand the right time to implement advance care planning. | ① | ② | ③ | ④ | ⑤ |

**SECTION 4**

Have you completed advance directives (ADs) for yourself?

o Yes

o No

Has anyone in your family completed advance directives (ADs)?

o Yes

o No

**Please answer the following questions considering your work over the past year:**

Have you cared for a patient who has had advance directives? (ADs)?

o Yes

o No

Have you cared for a patient who has had an advance care plan?

o Yes

o No

Do you believe that discussing/participating in discussions of advance care planning with the patient is part of your duty?

o Yes

o No

o I don’t know

If you answered No, who do you think should be responsible for this task?
o Physiotherapist
o Nurse
o Physician
o General practitioner
o Resident physician
o Psychologist
o Specialist caring for the patient
o Other – please specify __________________________________________________

Do you believe that participating in discussions about advance care planning with the patient is part of your duty?
o Yes
o No
o I don’t know

Roughly estimating, can you indicate the percentage of patients in your healthcare setting who are offered a discussion on advance care planning, considering only those with whom it would be appropriate to discuss it?
o More than 75%
o Between 51% and 75%
o Between 25% and 50%
o Less than 25%
o None
o Not applicable – please specify why ______________________________________

Have you ever personally discussed or taken part in a discussion about advance care planning with a patient?
o Yes
o No

If No, please go straight to Section 5

When you discuss or participate in the discussion of advance care planning, who usually initiates the discussion?
o The patient
o Family member/Caregiver

o Myself
o Other – please specify ______________________________________

How often are the patient’s family members/caregivers involved in the discussion of advance care planning?
o More than 75% of the time
o Between 51% and 75% of the time
o Between 25% and 50% of the time
o Less than 25% of the time
o Never
o I don’t know

How often are members of the healthcare team, in addition to the physician, involved in the discussion of advance care planning?
o More than 75% of the time
o Between 51% and 75% of the time

o Between 25% and 50% of the time

o Less than 25% of the time
o Never
o I don’t know

Which professionals are most frequently involved? You may select more than one answer.
▢ Physiotherapist
▢ Nurse
▢ Physician
▢ Resident physician / Physician in specialist training
▢ Psychologist
▢ Other – please specify ______________________________________

How often is advance care planning documented in the patient’s medical record/electronic health record?
o More than 75% of the time
o Between 51% and 75% of the time
o Between 25% and 50% of the time
o Less than 25% of the time
o Never
o I don’t know

**SECTION 5**

Did you complete your university studies (degree, specialization) before or after 2018?
o Before 2018
o In 2018 or later
o Still in progress

Have you received specific training or education on advance care planning?
o Yes, during university studies – theoretical training only
o Yes, during university studies – both theoretical and practical training
o Yes, after completing university studies – theoretical training only
o Yes, after completing university studies – both theoretical and practical training
o No

Have you received specific training or education on palliative care?
o Yes, through hours/days of training (e.g., lectures, seminars, conferences, refresher courses)
o Yes, through structured program (e.g., postgraduate education or master's programs in palliative care)
o No

Do you believe that there are obstacles to advance care planning in your healthcare organization?
o Yes
o No

If you answered Yes, what are those obstacles?

In your opinion, what could help or facilitate the implementation of advance care planning?

Has the content of Law 219/2017 regarding advance care planning been implemented in your healthcare organization with specific procedures? You may select more than one answer.
▢ Yes, at the local health trust level
▢ Yes, at the unit/service level
▢ Yes, other – please specify ______________________________________
▢ No
▢ I don’t know

After completing this questionnaire, would you be interested exploring advance care planning further?
o Yes, theoretical training
o Yes, practical training
o Yes, both theoretical and practical training
o No

Which of the following is your main main area of work/specialization?

How long have you worked in your current field? ___ years

Where does your work take place primarily?
o Outpatient clinic
o Inpatient ward
o Other – please specify ______________________________________

Approximately how many patients do you personally care for in a week? ___

If you were to estimate, how many of your patients do you expect could die in the next 12 months?
o More than 75%
o Between 51% and 75%
o Between 26% and 50%
o Less than 25%
o None
o Not applicable – please specify the reason ______________________________________

Considering the characteristics of the patients you care for, do you think advance care planning is a tool:
o Very useful
o Useful
o Slightly useful
o Not useful at all

How important are your religious or philosophical beliefs in your professional behavior regarding advance care planning?
o Very important
o Important
o Slightly important
o Not important at all

You may use the space below to provide any comments or clarifications regarding your answers:

______________________________________________________________________________________________________
