# Supplementary material for: Advance care planning knowledge, attitudes, and experiences among hospital healthcare professionals: A survey
Source: Palliat Support Care. 2026 Feb 19;24:e61. doi: 10.1017/S1478951526101874 (PMC13166266; doi:10.1017/S1478951526101874)
Supplement: Macchiarelli et al. supplementary material 2 — Macchiarelli et al. supplementary material [file S1478951526101874sup002.docx]

**Advance care planning knowledge, attitudes, and experiences among hospital healthcare professionals: a survey**

**Supplementary Table 1.** Additional results concerning participants’ characteristics and experiences with advance care planning (N=724).

|  |  | All  (724) | Physicians  (259) | Residents  (86) | Nurses  (339) | Physioth.  (40) | *p* |
| --- | --- | --- | --- | --- | --- | --- | --- |
| Professional experience (years) | Mean (SD) | 15.8±11.2 | 18.1±10.5 | 3.0±3.5 | 16.5±11.0 | 22.8±10.9 | <0.001 |
| Experience in the current field (years) | Mean (SD) | 10.8±10.42 | 14.8±10.4 | 2.4±2.6 | 9.3±9.6 | 16.3±12.5 | <0.001 |
| Main care delivery setting % (n) | Ward  Outpatient Clinic  Other | 67.40 (488)  14.92 (108)  17.68 (128) | 51.7 (134)  20.1 (52)  28.2 (73) | 57 (49)  19.7 (17)  23.3 (20) | 82 (278)  9.4 (32)  8.5 (29) | 67.5 (27)  17.5 (7)  15.0 (6) | <0.001 |
| Number of patients cared for during a week | Mean (SD) | 27.0±25.6 | 32.3±26.3 | 21.2±16.5 | 24.9±27.5 | 23.9±12.0 | <0.001 |

**Supplementary Table 2.** Percentage of participants’ correct answers to the knowledge section (N=547).

|  | All  (547) | Physicians  (201) | Residents  (57) | Nurses  (261) | Physioth.  (28) | *p* |
| --- | --- | --- | --- | --- | --- | --- |
| ACP may be carried out in relation to the progression of chronic or disabling diseases or diseases with poor prognosis % (n) | 98.2 (537) | 99.5 (200) | 100.0 (57) | 96.5 (252) | 100 (28) | 0.061 |
| ACP can be done without patient signing up to an advance directive % (n) | 84.3 (461) | 93.5 (188) | 80.1 (46) | 80.9 (211) | 57.1 (16) | <0.001 |
| Within ACP the patient expresses their wills regarding the care plan proposed and may indicate the name of a fiduciary % (n) | 97.3 (532) | 97.5 (196) | 96.5 (55) | 97.7 (255) | 92.7 (26) | 0.494 |
| The healthcare team must always adhere to the ACP should the patient be in a condition of incapacity % (n) | 77.7 (425) | 79.6 (160) | 68.4 (39) | 78.5 (205) | 75.0 (21) | 0.324 |
| ACP has no legal value % (n) | 83.0 (454) | 82.1 (165) | 80.7 (46) | 82.8 (216) | 96.4 (27) | 0.270 |
| Good communication skills are essential for ACP discussion % (n) | 97.6 (534) | 98.5 (198) | 100 (57) | 96.9 (253) | 100 (28) | 0.149 |
| ACP must be documented as a public act or as a notarized private agreement % (n) | 41.5 (227) | 52.7 (106) | 26.3 (15) | 36.4 (95) | 39.3 (11) | <0.001 |
| Patient consent is necessary to involve family/caregivers in ACP % (n) | 93.0 (509) | 92.5 (186) | 96.5 (55) | 93.1 (243) | 89.3 (25) | 0.628 |
| ACP can be updated as the disease progresses upon the patient’s request or the physician’s recommendation % (n) | 98.9 (541) | 98.5 (201) | 100 (57) | 100 (261) | 100 (28) | 0.759 |

AD: advance directives; ACP: advance care planning.

**Supplementary Table 3.** Additional results concerning participants’ experiences with advance care planning during the last year.

| **When you discuss/participate in the discussion of advance care planning, who usually initiates the discussion?** | | | | | | | | | | | |
| --- | --- | --- | --- | --- | --- | --- | --- | --- | --- | --- | --- |
| % (N) | **All**  **(250)** | **Physician**  **(148)** | | | | **Resident**  **(21)** | **Nurse**  **(72)** | | | **Physioth.**  **(9)** | *p* |
| Myself | 47.2% (118) | 65.5% (97) | | | | 38.1% (8) | 16.7% (12) | | | 11.1% (1) | < 0.001 |
| The patient | 10.8% (27) | 8.8% (13) | | | | 4.8% (1) | 18.1% (13) | | | 0.0% (0) |  |
| Family member/Caregiver | 9.2% (23) | 6.8% (10) | | | | 9.5% (2) | 13.9% (10) | | | 11.1% (1) |  |
| Other | 32.8% (82) | 18.9% (28) | | | | 47.6% (10) | 51.4% (37) | | | 77.8% (7) |  |
| Other (multiple answers were possible) | | | | | |  | | | | | |
| N Profession | | | | | | Answer (N) | | | | | |
| 28 Nurses, 9 Resident, 5 Physiotherapists, 2 Physician | | | | | | Physician (44) | | | | | |
| 7 Physicians, 4 Nurses, 1 Physiotherapist | | | | | | Palliative Care Physician (12) | | | | | |
| 7 Physicians, 1 Resident, 1 Nurse, 1 Physiotherapist | | | | | | Palliative Care Unit (10) | | | | | |
| 4 Physicians, 1 Nurse | | | | | | General Practitioner (5) | | | | | |
| 3 Nurses, 2 Physicians | | | | | | Equipe/Care Unit (5) | | | | | |
| 2 Physicians | | | | | | Ward Manager (2) | | | | | |
| 3 Physicians, 2 Nurse, 1 Resident | | | | | | Other (6) | | | | | |
| **How often are the patient’s family members/caregivers involved in the discussion of advance care planning?** | | | | | | | | | | | |
| <25% | 3.2% (8) | | 1.3% (2) | | 0.0% (0) | | | 8.3% (6) | | 0.0% (0) | 0.038 |
| 25-50% | 6.0% (15) | | 2.7% (4) | | 4.8% (1) | | | 12.5% (9) | | 11.1% (1) |  |
| 51-75% | 19.2% (48) | | 19.6% (29) | | 23.8% (5) | | | 15.3% (11) | | 33.3% (3) |  |
| >75% | 65.2% (163) | | 68.9% (102) | | 71.4% (15) | | | 57.0% (41) | | 55.6% (5) |  |
| **How often are other members of the healthcare team involved in the discussion of advance care planning?** | | | | | | | | | | | |
| Don’t Know | 7.6% (19) | | 6.8% (10) | | 0.0% (0) | | | 9.7% (7) | | 22.2% (2) | 0.010 |
| Never | 4.0% (10) | | 4.0% (6) | | 9.5% (2) | | | 2.8% (2) | | 0.0% (0) |  |
| <25% | 14.8% (37) | | 10.8% (16) | | 9.5% (2) | | | 22.2% (16) | | 33.3% (3) |  |
| 25-50% | 13.6% (34) | | 10.8% (16) | | 38.1% (8) | | | 13.9% (10) | | 0.0% (0) |  |
| 51-75% | 20.8% (52) | | 20.9% (31) | | 14.3% (3) | | | 22.2% (16) | | 22.2% (2) |  |
| >75% | 39.2% (98) | | 46.6% (69) | | 28.6% (6) | | | 29.2% (21) | | 22.2% (2) |  |
| **Which healthcare professionals are most frequently involved?** (multiple answers were possible) | | | | | | | | | | | |
| N Profession | Answer (N) | | |  | | | | |  | | |
| 108 Physicians, 55 Nurses, 17 Residents, 7 Physiotherapists | Physician (187) | | |  | | | | |  | | |
| 39 Physicians, 26 Nurses, 13 Residents, 3 Physiotherapists | Resident (81) | | |  | | | | |  | | |
| 91 Physicians, 43 Nurses, 7 Residents, 5 Physiotherapists | Nurse (146) | | |  | | | | |  | | |
| 14 Physicians, 7 Nurses, 3 Physiotherapists, 1 Resident | Physiotherapist (25) | | |  | | | | |  | | |
| 46 Physicians, 27 Nurses, 3 Residents, 2 Physiotherapists | Psychologist (78) | | |  | | | | |  | | |
| 20 Physicians, 7 Nurses, 4 Residents, 2 Physiotherapists | Other (33) → | | | N Profession | | | | | Answer (N) | | |
|  |  | | | 10 Physicians, 1 Nurse | | | | | Palliative Care Unit (11) | | |
|  |  | | | 4 Physicians, 2 Resident, 1 Nurse | | | | | Palliative Care Physician (7) | | |
|  |  | | | 2 Physicians, 1 Nurse, 1 Physiotherapist | | | | | Social Worker (4) | | |
|  |  | | | 6 Nurses, 4 Physicians, 3 Residents, 2 Physiotherapists | | | | | Other (15) | | |
